# Hospital Festivals and Meetings

**Published:** 1895-02-09

**Authors:** 


### HOSPITAL FESTIVALS AND MEETINGS.

### ROYAL MATERNITY CHARITY.

This charity held its annual general meeting on January 29th at 31, Finsbury Square, E.C., Mr. C. Barham, C.C., in the chair. The report showed that 4,390 poor women were attended during the past year. The staff of district surgeons and midwives had been increased in numbers and efficiency, and in several districts midwives had been supplied who could communicate with the many foreign patients in their own tongues. The chairman earnestly appealed for additional funds to carry on the good and useful work of the charity, one of the most deserving in the kingdom. The number of poor women helped by it in 1894 reached a larger total than in any one year since 1825.

### THE HOSTEL OF ST. LUKE.

The annual meeting of subscribers to this charity was held last week at the Church House, Dean's Yard, Westminster, the Bishop of London presiding. The nursing home, or hostel, which is intended for the benefit of poor clergy, is only in the third year of its existence, and has been the means of rendering most valuable assistance in many distressing cases during the past twelve months. Three classes of patients are received, those who can pay, those who cannot, and out-patients. Since August last the hostel has been established at 16, Nottingham Place, Marylebone Road, a house well suited for the purpose, and which has been adequately fitted up. The Bishop of London, who moved the adoption of the report, said continued and increased support was much needed, because the charity was one likely to grow largely. Bishop Selwyn, Canon Sir J. E. Phillips, and Canon Utterton also spoke on behalf of the institution, and the following motion was proposed and warmly seconded: "That the Hostel of St. Luke, being a nursing home for the use of those clergy of the Church of England, their wives or widows and children, whose circumstances do not permit of their obtaining adequate medical or surgical treatment at their own houses, and providing all the advantages of hospital nursing and care without the disadvantages of large public wards, deserves the hearty support and sympathy of all Church people."

### GENERAL LYING-IN HOSPITAL, YORK ROAD, LAMBETH.

The annual general meeting of the governors and subscribers of this hospital was held on Wednesday, January 29th. The chair was taken, in the absence of the president, Lord Darnley, unable through illness to attend, by the Hon. Alan de Tatton Egerton, M.P. The report, which was read by the chairman, showed that there had been during the past year an excess of expenditure over income of rather over £200, a deficit which the committee were anxious to make up as soon as possible. Thirty-five midwives and seventy-five nurses had been admitted for training, and the committee especially acknowledged the help rendered them in this connection by the examinations of the London Obstetrical Society, help of which they regretted to find it possible they might now be deprived. They observed with pleasure that a scheme had been prepared for the statutory registration of midwives. The medical report, read by Dr. Herman, gave satisfactory evidence of the year's work. More patients had been treated than ever before, whilst the number of deaths was reduced.

Dr. Boxall, in moving the adoption of the report, said he had known the hospital well for many years, and he considered that its work was carried on with efficiency and the greatest economy possible. He understood that some of the wards needed re-flooring, and that he should be glad to make himself responsible for the carrying out of this improvement in one of them, in memory of a relative, and he trusted that others might come forward with similar offers.

In re-electing members of the committee, &c., it was decided to ask Lord Darnley to continue as president for the ensuing year, and Lord Saye and Sele having resigned the office of treasurer, Mr. de Tatton Egerton consented to fill that post temporarily. The usual votes of thanks followed.

